# Computed Tomography Optic Nerve Sheath Diameter-to-Eyeball Transverse Diameter Ratio as a Novel Noninvasive Parameter for Prognostication in Traumatic Brain Injury

**DOI:** 10.7759/cureus.68297

**Published:** 2024-08-31

**Authors:** Praveen K Sharma, Paarthipan Natarajan, Govindarajan BR, Karthik Krishna Ramakrishnan, Arun Aram, Sakthi Ganesh Subramonian

**Affiliations:** 1 Department of Radiology, Saveetha Medical College and Hospital, Saveetha Institute of Medical and Technical Sciences, Saveetha University, Chennai, IND

**Keywords:** noninvasive intracranial pressure, traumatic brain injuries, optic nerve sheath diameter, intracranial hypertension, glasgow coma scale, brain injuries, traumatic, optic nerve, computed tomography

## Abstract

Background

Traumatic brain injury (TBI) remains a foremost cause of death and disability globally, with elevated intracranial pressure (ICP) being a crucial factor in patient outcomes. While invasive monitoring is the gold standard for assessing ICP, it carries risks and is not always feasible. This study proposes a novel noninvasive parameter using computed tomography (CT) imaging.

Aims and objectives

The study aims to determine the efficacy of the optic nerve sheath diameter (ONSD)-to-eyeball transverse diameter (ETD) ratio from CT scans in predicting TBI patients' prognosis. The primary objective is to study the ONSD/ETD ratio's efficacy in assessing TBI's severity. The secondary objective is to correlate the ONSD/ETD ratio with the Glasgow Coma Scale (GCS) and Rotterdam computed tomography scoring (RCTS) and assess its clinical benefit.

Materials and methods

This combined retrospective and prospective analytical study included 308 consecutive patients who underwent CT imaging for TBI at a tertiary care center with a dedicated trauma and neurosurgical unit. We evaluated bilateral ONSD and ETD using axial CT scans. The ONSD/ETD ratio correlated with the GCS, RCTS, and clinical outcomes.

Results

The cut-off values for elevated ICP were ONSD of >5.17 mm, ETD of <22.2 mm, and ONSD/ETD ratio of >0.21. Variables between GCS (<12 and >12) and the ONSD/ETD ratio (<0.21 and >0.21) were statistically significant (chi-square {χ^2^} = 18.52, p = 0.000). The ONSD shows a strong positive correlation with RCTS (r = 0.82, p = 0.01), ETD shows a moderate negative correlation with RCTS (r = -0.50), and the ONSD/ETD ratio shows a strong negative correlation with GCS (r = -0.783, p = 0.01). The area under the curve for the ONSD/ETD ratio (0.920) was higher than that for ONSD (0.932) and ETD (0.490). The ONSD/ETD ratio's sensitivity, specificity, positive predictive value, and negative predictive value were 100%, 95.6%, 72.0%, and 100%, respectively, demonstrating that it is an excellent predictor of raised ICP.

Conclusion

The CT-ONSD/ETD ratio correlates with the severity of TBI as assessed by GCS and RCTS. It could serve as a noninvasive parameter for monitoring ICP and guiding the need for sequential CT in TBI patients, potentially aiding in prognostication and clinical management.

## Introduction

Traumatic brain injury (TBI) is one of the foremost reasons for morbidity, mortality, disability, dependence, and socioeconomic loss in India and worldwide [1]. Every year, nearly one million people in India lose their lives, and 1.5-2 million become disabled. Road traffic accidents (RTA) (60%), falls (20%-25%), and violence (10%) are the causes of TBIs [2]. The prognosis of a person with TBI depends on several factors, such as their age and gender, the severity of the injury as measured by the Glasgow Coma Scale (GCS) and intracranial pressure (ICP), the motor score, the time of diagnosis, any secondary injuries, their use of anticoagulants at the same time, their pupil reactivity, their blood levels of specific proteins, and any other comorbidities [3]. Post-TBI disability is a significant concern in providing optimal care to TBI patients. Elevated ICP (above 20 mm Hg) is one of the essential factors for morbidity and mortality after TBI and causes secondary damage to the ischemic brain [4]. High ICP is a crucial indicator and pivotal to managing the worse outcomes and mortality of severe TBI patients in nonintervention. The timely diagnosis and control of elevated ICP could prevent detrimental effects such as herniation and decrease mortality [5].

The gold criterion for diagnosing raised ICP is invasive external ventricular device (EVD) monitoring. With coagulopathies, EVD contraindicates intraventricular catheter placement [6]. In these cases, acute cerebrospinal fluid drainage can displace the cerebral structures and, in severe cases, even provoke sub-incarceration. EVD monitoring necessitates a skilled, holistic technical team; equipment; and technical challenges. Like inadequate ventricular width, it is more of a theoretical aspiration than a real-world solution in an emergency room (ER) setting [7], and it can lead to problems after the procedure, such as infection, bleeding, and misplacement [8]. Alternative techniques include pneumatic sensors, strain gauges, fiber-optic devices, and micro-transducer ICP monitoring systems. The BEST-TRIP trial questioned the necessity of ICP monitor placement, as it found no difference in output with ICP monitoring [9]. The Brain Trauma Foundation recommended ICP monitoring in the TBI (GCS of 3-8, abnormal computed tomography {CT} of the head, age of >40, posturing or systolic blood pressure of <90, and standard CT of the head). The foundation recommends "ICP monitoring to reduce two-week post-injury and in-hospital mortality" [10].

This article will carefully examine the current research and clinical data to determine whether the optic nerve sheath diameter (ONSD)-to-eyeball transverse diameter (ETD) ratio is valid, reliable, and helpful in predicting the outcome of TBI. We hypothesize that the ONSD/ETD ratio will significantly correlate with specific severity indicators and outcome measures and suggest that it could be helpful in the current tools used to predict TBI outcomes.

## Materials and methods

This study received approval from the Saveetha Medical College and Hospital (SMCH) Local Biomedical Research Ethics Committee. We obtained verbal informed consent from all prospective subjects in the study, making provisions to communicate in the aboriginal language if necessary. The ethical committee approval details are as follows: number 003/02/2021/IEC/SMCH, dated 17/02/2021. The study used a retrospective and a prospective analytical study design and looked at 308 consecutive patients with serial computed tomography (CT) imaging for TBI. We conducted the study at SMCH, Thandalam, a tertiary care center with a dedicated neurosurgical and trauma unit. The study retrospectively covered the period from 2018 to 2020 using secondary data from CT of the brain studies, a routine part of the institution's trauma protocol, and primary data prospectively from 2021 using non-probability convenience sampling methods.

ONSD and ETD measurement protocol

**Figure 1:**
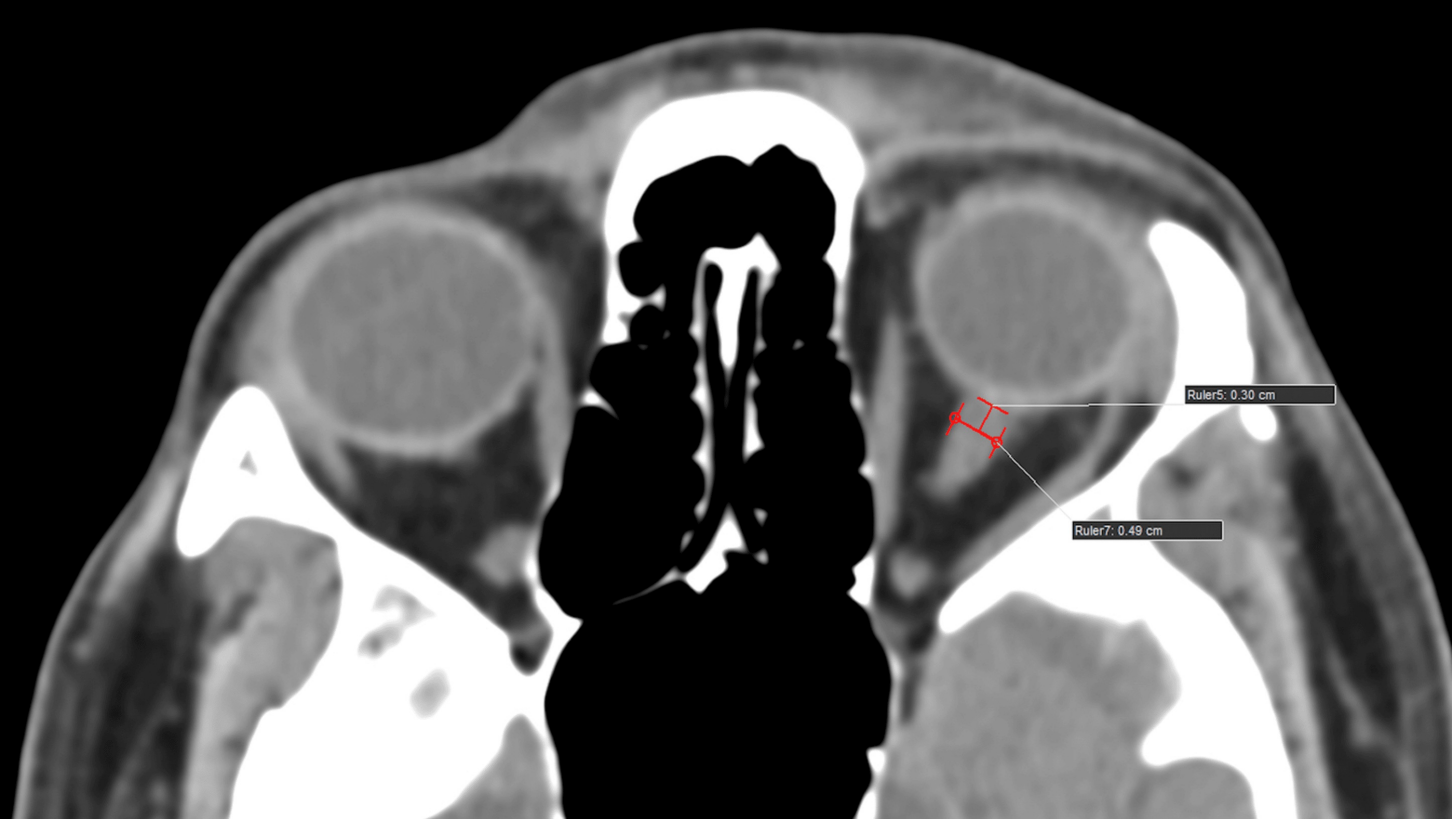
An average healthy subject/individual. A computed tomography (CT) scan of the brain (axial section, 1 mm slice thickness, standard mediastinum algorithm, window level of 10 Hounsfield units {HU} and width of 300 HU) shows optic nerve sheath diameter (ONSD) measured transversely (3 mm behind the globe).

**Figure 2:**
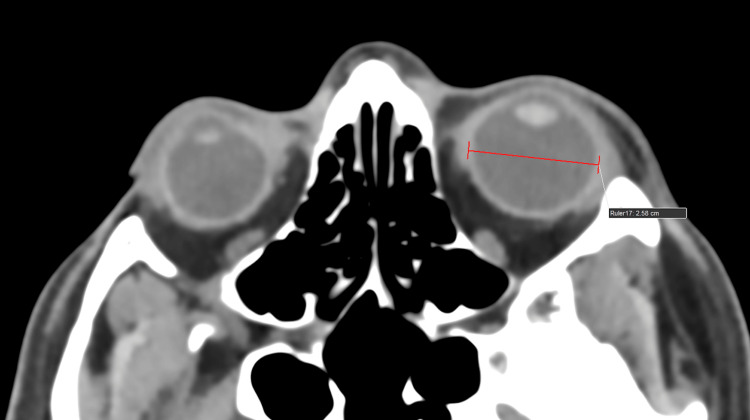
An average healthy subject/individual. A computed tomography (CT) scan of the brain (axial section, 1 mm slice thickness, standard mediastinum algorithm, window level of 10 Hounsfield units {HU} and width of 300 HU) shows eyeball transverse diameter (ETD) measured transversely (from sclera to sclera).

As part of the institution's protocol, all head trauma patients underwent CT scanning (Philips 128 slice, Ingenuity, Philips, Amsterdam, Netherlands) using a standard mediastinum algorithm (window level of 10 Hounsfield units {HU} and width of 300 HU). Primary data obtained from bilateral ONSD measured 3 mm behind the eyeball in the CT axial plane (1 mm slice thickness). Bilateral ETD was measured transversely from sclera to sclera in the CT axial plane (1 mm slice thickness) (Figures 1, 2).

We obtained measurements of ONSD and ETD by adhering to a rigid protocol established to minimize interobserver variability. Average ONSD and ETD were also used in our statistical analysis to help reduce the laterality effects of lesions. The GCS was assessed clinically, and CT evaluated the Rotterdam computed tomography scoring (RCTS). Bias was eliminated by blinding RCTS to ONSD using the Medsynapse Radiology Picture Archiving and Communication Software (PACS) (Medsynaptic Pvt. Ltd., Pune, India).

Variables analyzed

In this study, the ONSD measured 3 mm behind the eyeball is used as an indicator to predict elevated ICP or intracranial hypertension (ICH). A cut-off value of 5.17 mm was established, where values below this threshold are considered normal, and values above predict elevated ICP or ICH. Reference values indicate that ONSD ranges from 3.65 mm to 5.17 mm within the intra-orbital space in healthy individuals, with no significant differences observed between gender and age groups [11]. In this study, the ETD measured from sclera to sclera has a cut-off value of 22.2 mm, with measurements above considered normal and those below predicted elevated ICP or ICH. Reference values suggest that in healthy individuals, the ETD averages around 24.156 ± 1.9 mm in the right eyeball and 24.324 ± 1.9 mm in the left eyeball [12]. The ONSD/ETD ratio, a calculated index of ONSD divided by ETD, has a cut-off value of 0.21 in this study, where values below are considered normal, and values above predict elevated ICP or ICH. Reference values indicate that this ratio typically has a mean of 0.19 with a standard deviation of 0.01-0.02, while the study observed a mean of 0.24 ± 0.03 [11,13].

The RCTS and GCS further assess the severity of TBI in patients. In this study, RCTS scores of 2 and 3 indicate mild TBI, while scores of 4 and above are associated with severe TBI. Reference values categorize patients into mild and severe groups based on their RCTS scores [14]. For the GCS, a score above 12 is considered mild TBI, while a score below 12 indicates severe TBI. Reference values classify GCS scores from 13 to 15 as mild, 9 to 12 as moderate, and 3 to 8 as severe, providing a framework for evaluating patient outcomes about their initial neurological status [15].

Inclusion and exclusion criteria

The inclusion criteria for this study comprised all patients presenting with TBI due to road traffic accidents (RTA), falls, or assaults who underwent serial CT imaging follow-up, regardless of age and gender. We excluded from the study patients without a history of trauma, those with unilateral or bilateral ocular injuries, and those with unilateral or bilateral ocular nontraumatic pathologies such as pseudopapilledema. We excluded patients with malignancies, hydrocephalus, or low-quality imaging (due to artifacts). We also excluded patients who were not available for CT imaging, such as acutely moribund patients who succumbed to injuries within 24 hours of admission after initial CT and severe emergent patients who underwent immediate operative intervention after initial CT. The study's expected outcome is that the ONSD/ETD ratio will increase in TBI cases and mirror the rise in ICP, determining TBI severity. We anticipate a strong correlation between the ONSD/ETD ratio and the RCTS's ICP-based visual severity score, which could serve as an adjunct parameter. Additionally, the ONSD/ETD ratio combined with the RCTS may provide a congruent review of clinical severity at presentation with progression reflected by the GCS.

Data/statistical analysis

We meticulously added each data point to our study database using the Statistical Package for Social Sciences (SPSS) program version 21 (IBM SPSS Statistics, Armonk, NY). A descriptive analysis used frequency (number percent) as the expression for categorical characteristics. We used analytical statistics, the chi-square (χ^2^) test, and the p-value to assess significance after calculating the means for continuous variables. Correlations between variables of interest were determined using non-parametric correlations, specifically Spearman's rho test and R² linear (coefficient of determination). We calculated the GCS, RCTS, ONSD, ETD, and ONSD/ETD ratios to ascertain their sensitivity, specificity, and positive and negative predictive values. We computed the average or mean ONSD and ETD of both eyes. We primarily correlated the ONSD/ETD ratio with the radio-pathological severity and prognosis provided by RCTS to establish its potential role as an adjunct surrogate for raised ICP. The ONSD/ETD ratio also showed a secondary correlation with GCS, a measure of clinical severity. The correlation analysis encompassed these variables, along with gender and age. We used the chi-square test to determine significance, accepting a p-value of less than 0.05 as the threshold for statistical significance.

## Results

This study included 308 patients. We acquired retrospective secondary data from 200 CT brain studies conducted as part of the institution's trauma protocol from 2018 to 2020. We collected the primary data prospectively from 2021, using non-probability convenience sampling, and included 108 patients who met the inclusion criteria. The study population comprised 262 male patients (85.1%) and 46 female patients (14.9%).

The study population's baseline features revealed significant differences in essential measurements and demographic data. The age distribution was as follows: 8.8% were under 20 years old, 39.6% were aged 21-40 years, 36% were aged 41-60 years, 13.6% were aged 61-80 years, and 1.9% were over 81 years old. The frequencies of ONSD measurements were 14.6% for values of <5.17 mm and 85.4% for values of >5.17 mm. For ETD, the frequencies were 82.8% for values of >22.2 mm and 17.2% for values of <22.2 mm. The ONSD/ETD ratio showed that 7.1% of patients had values of <0.21, while 92.9% had values of >0.21 (Table 1).

**Table 1:** Frequency (n) and percentage (%) of variables (i) optic nerve sheath diameter (ONSD), (ii) eyeball transverse diameter (ETD), and (iii) ONSD/ETD ratio in traumatic brain injury (TBI).

Serial number	Variables	mm	Frequency (n)	%
i	ONSD	<5.17	45	14.6
		>5.17	263	85.4
ii	ETD	>22.2	255	82.8
		<22.2	53	17.2
iii	ONSD/ETD ratio	<0.21	22	7.1
		>0.21	286	92.9

The relationship between GCS (<12 versus >12) to ONSD (<5.17 mm versus >5.17 mm), ETD (>22.2 mm versus <22.2 mm), and the ONSD/ETD ratio (<0.21 versus >0.21) was analyzed. The chi-square analysis revealed the following results: for ONSD, the chi-square value was 39.9, with a p-value of 0.000, indicating a statistically significant association. For ETD, the chi-square value was 2.132 with a p-value of 0.153, which was not statistically significant. The ONSD/ETD ratio showed a chi-square value of 18.52 with a p-value of 0.000, indicating a statistically significant association (Tables 2-4).

**Table 2:** Association between Glasgow Coma Scale (GCS) and optic nerve sheath diameter (ONSD) in traumatic brain injury (TBI). *The correlation is significant.

Variables	ONSD < 5.17 mm, n (%)	ONSD > 5.17 mm, n (%)	Chi-square	P-value
GCS < 12	0 (0%)	102 (100%)	39.9	0.000*
GCS > 12	45 (21.8%)	161 (78.2%)		

**Table 3:** Association between Glasgow Coma Scale (GCS) and eyeball transverse diameter (ETD) in traumatic brain injury (TBI).

Variables	ETD > 22.2 mm, n (%)	ETD < 22.2 mm, n (%)	Chi-square	P-value
GCS < 12	89 (87.2%)	13 (12.8%)	2.132	0.153
GCS > 12	166 (80.5%)	40 (19.5%)		

**Table 4:** Association between Glasgow Coma Scale (GCS) and the ONSD/ETD ratio in traumatic brain injury (TBI). *The correlation is significant. ONSD, optic nerve sheath diameter; ETD, eyeball transverse diameter

Variables	ONSD/ETD ratio < 0.21, n (%)	ONSD/ETD ratio > 0.21, n (%)	Chi-square	P-value
GCS < 12	0 (0%)	102 (100%)	18.52	0.000*
GCS > 12	22 (10.6%)	184 (89.4%)		

The correlation between variables of interest revealed the following relationships: (a) The mean ONSD and RCTS showed a strong positive correlation, with a correlation coefficient of 0.82, significant at the 0.01 level (two-tailed); (b) the mean ETD and RCTS demonstrated a moderate negative correlation, with a correlation coefficient of -0.50, but this correlation was statistically non-significant; and (c) the mean ONSD/ETD ratio and GCS showed a strong negative correlation, with a correlation coefficient of -0.783, significant at the 0.01 level (two-tailed) (Tables 5-7).

**Table 5:** Correlation between the mean optic nerve sheath diameter (ONSD) and Rotterdam computed tomography scoring (RCTS). **Correlation is significant at 0.01 level (two-tailed), with a strong positive correlation.

Variable 1	Variable 2	Correlation
Mean ONSD	Mean ONSD	1
Mean ONSD	RCTS	0.82**

**Table 6:** Correlation between the mean eyeball transverse diameter (ETD) and Rotterdam computed tomography scoring (RCTS). Note: The correlation is non-significant, with a moderate negative correlation.

Variable 1	Variable 2	Correlation
Mean ETD	Mean ETD	1
Mean ETD	RCTS	-0.50

**Table 7:** Correlation between the mean ONSD/ETD ratio and Glasgow Coma Scale (GCS). **Correlation is significant at 0.01 level (two-tailed), with a strong negative correlation. ONSD, optic nerve sheath diameter; ETD, eyeball transverse diameter

Variable 1	Variable 2	Correlation
Mean ONSD/ETD ratio	Mean ONSD/ETD ratio	1
Mean ONSD/ETD ratio	GCS	-0.783**

The non-parametric correlation (Spearman's rho) between the GCS and the RCTS has a correlation coefficient of -0.931 and a significant (two-tailed) value of 0.000 (Table 8).

**Table 8:** Correlation between the Glasgow Coma Scale (GCS) and the Rotterdam computed tomography scoring (RCTS). N: total number of patients

Variable 1	Variable 2	Correlation type	Correlation coefficient	Significance (two-tailed)	N
GCS	RCTS	Spearman's rho	-0.931	0.000	308

**Figure 3:**
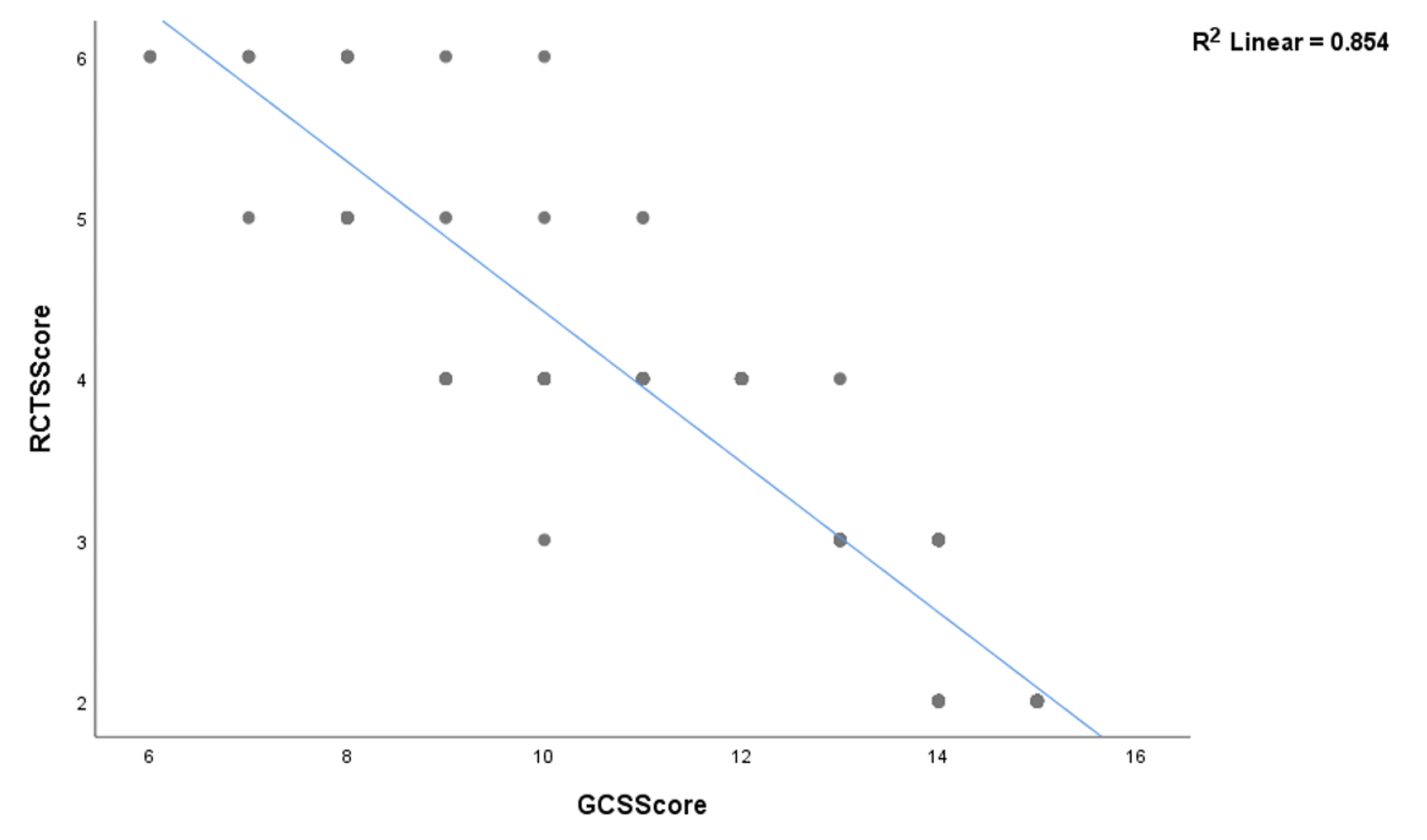
R² linear (coefficient of determination) of the Glasgow Coma Scale (GCS) and Rotterdam computed tomography scoring (RCTS) is 0.854.

The R² linear (coefficient of determination) of GCS and RCTS is 0.854 (Figure 3).

**Figure 4:**
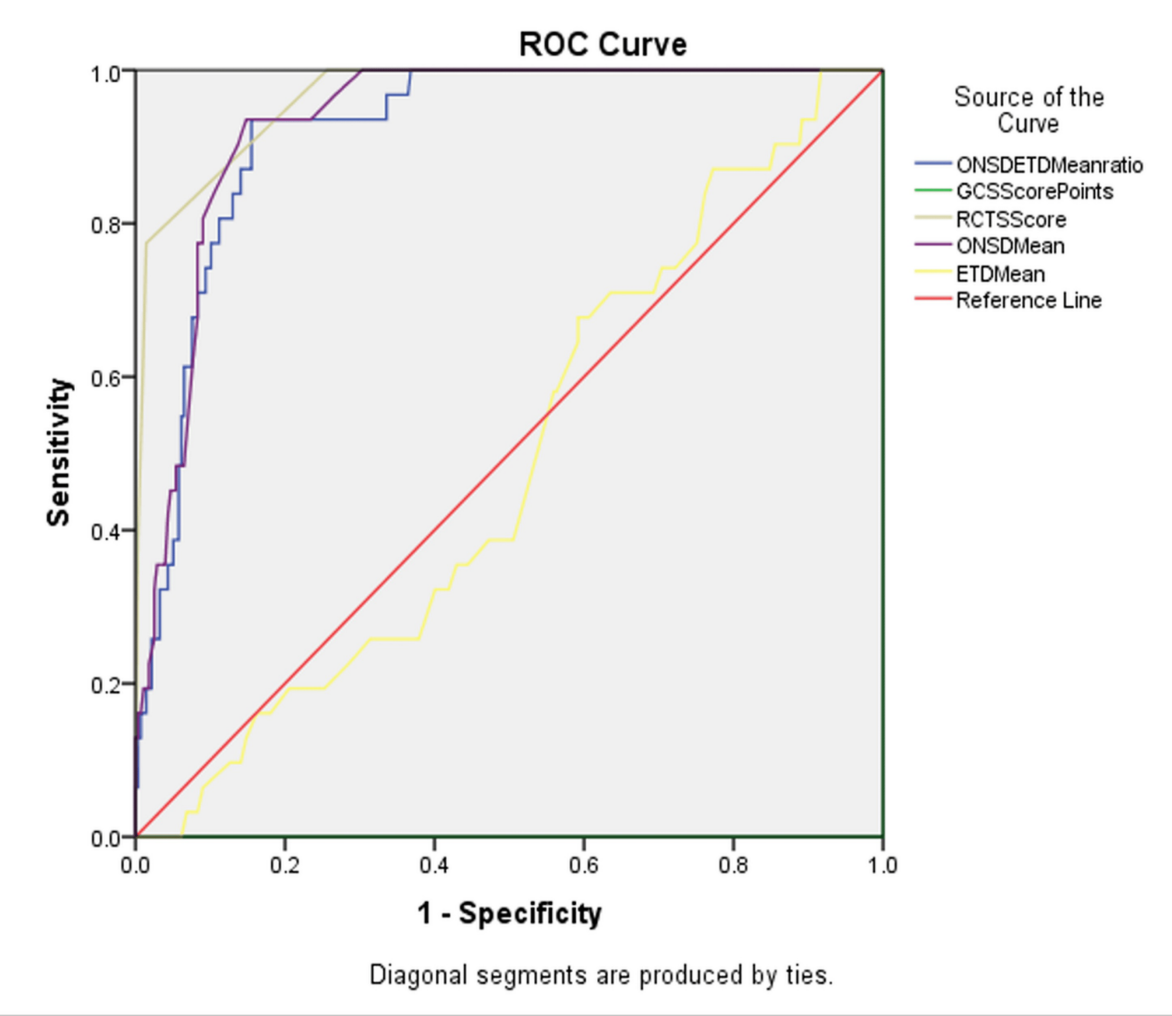
Receiver operating characteristic (ROC) curves for various diagnostic parameters in traumatic brain injury (TBI), showing the trade-off between sensitivity and specificity for each parameter. ONSD, optic nerve sheath diameter; ETD, eyeball transverse diameter; GCS, Glasgow Coma Scale; RCTS, Rotterdam computed tomography scoring

The sensitivity, specificity, positive predictive value, and negative predictive value were as follows: (a) GCS: 100%, 86.2%, 44.9%, and 100%, respectively; (b) RCTS: 100%, 74.3%, 30.3%, and 100%, respectively; (c) ONSD: 100%, 83.7%, 40.7%, and 100%, respectively; (d) ETD: 87.0%, 79.4%, 32.1%, and 98.2%, respectively; and (e) ONSD/ETD ratio: 100%, 95.6%, 72.0%, and 100%, respectively (Figure 4 and Table 9).

**Table 9:** Sensitivity, specificity, positive predictive value, and negative predictive value of the Glasgow Coma Scale (GCS), Rotterdam computed tomography score (RCTS), optic nerve sheath diameter (ONSD), eyeball transverse diameter (ETD), and ONSD/ETD ratio.

Variables	Sensitivity	Specificity	Positive predictive value	Negative predictive value
GCS	100%	86.2%	44.9%	100%
RCTS	100%	74.3%	30.3%	100%
ONSD	100%	83.7%	40.7%	100%
ETD	87.0%	79.4%	32.1%	98.2%
ONSD/ETD ratio	100%	95.6%	72.0%	100%

The area under the curve for the GCS, RCTS, ONSD, ETD, and ONSD/ETD ratio are 0.000, 0.965, 0.932, 0.490, and 0.920, respectively (Table 10).

**Table 10:** The area under the curve of the Glasgow Coma Scale (GCS), Rotterdam computed tomography score (RCTS), optic nerve sheath diameter (ONSD), eyeball transverse diameter (ETD), and ONSD/ETD ratio.

Variables	The area under the curve
GCS (scale)	0.000
RCTS (score)	0.965
ONSD (mm)	0.932
ETD (mm)	0.490
ONSD/ETD ratio (mm/mm)	0.920

These values indicate an association between the variables, which is statistically significant.

Representative case presentation

**Figure 5:**
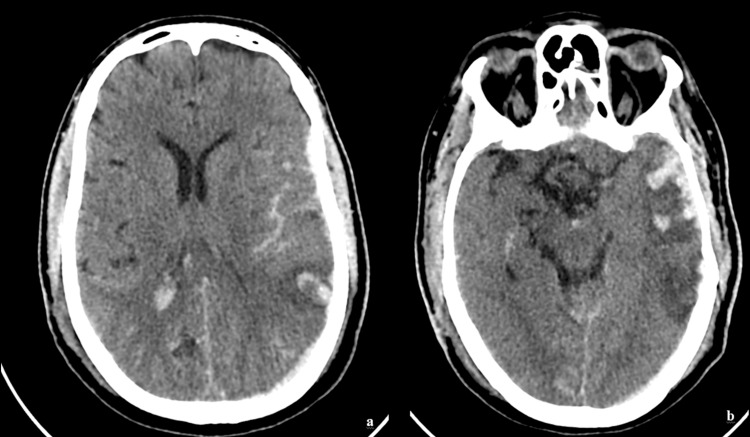
(a and b) A 55-year-old male who was involved in a road traffic accident and sustained a head injury presented with a Glasgow Coma Scale (GCS) of 7. The initial computed tomography (CT) scan of the brain (axial section, 1 mm slice thickness, standard mediastinum algorithm with a window level of 10 Hounsfield units {HU} and a width of 300 HU) shows a Rotterdam computed tomography score (RCTS) of 5, indicating severe traumatic brain injury (TBI).

**Figure 6:**
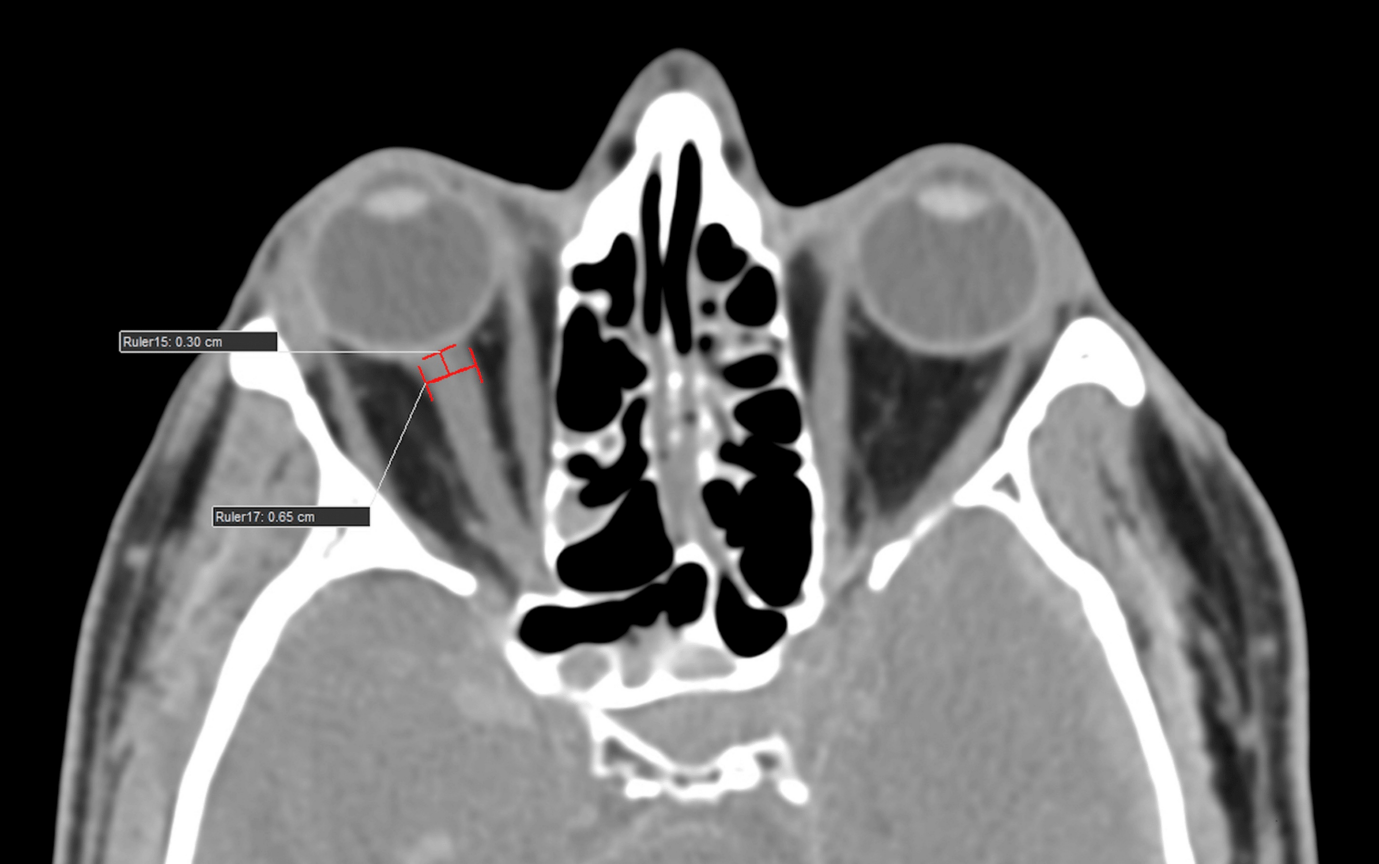
A 55-year-old male who was involved in a road traffic accident and sustained a head injury presented with a Glasgow Coma Scale (GCS) of 7. The initial computed tomography (CT) scan of the brain (axial section, 1 mm slice thickness, standard mediastinum algorithm with a window level of 10 Hounsfield units {HU} and a width of 300 HU) shows an optic nerve sheath diameter (ONSD) of 6.5 mm at 3 mm behind the globe, indicating severe traumatic brain injury (TBI).

**Figure 7:**
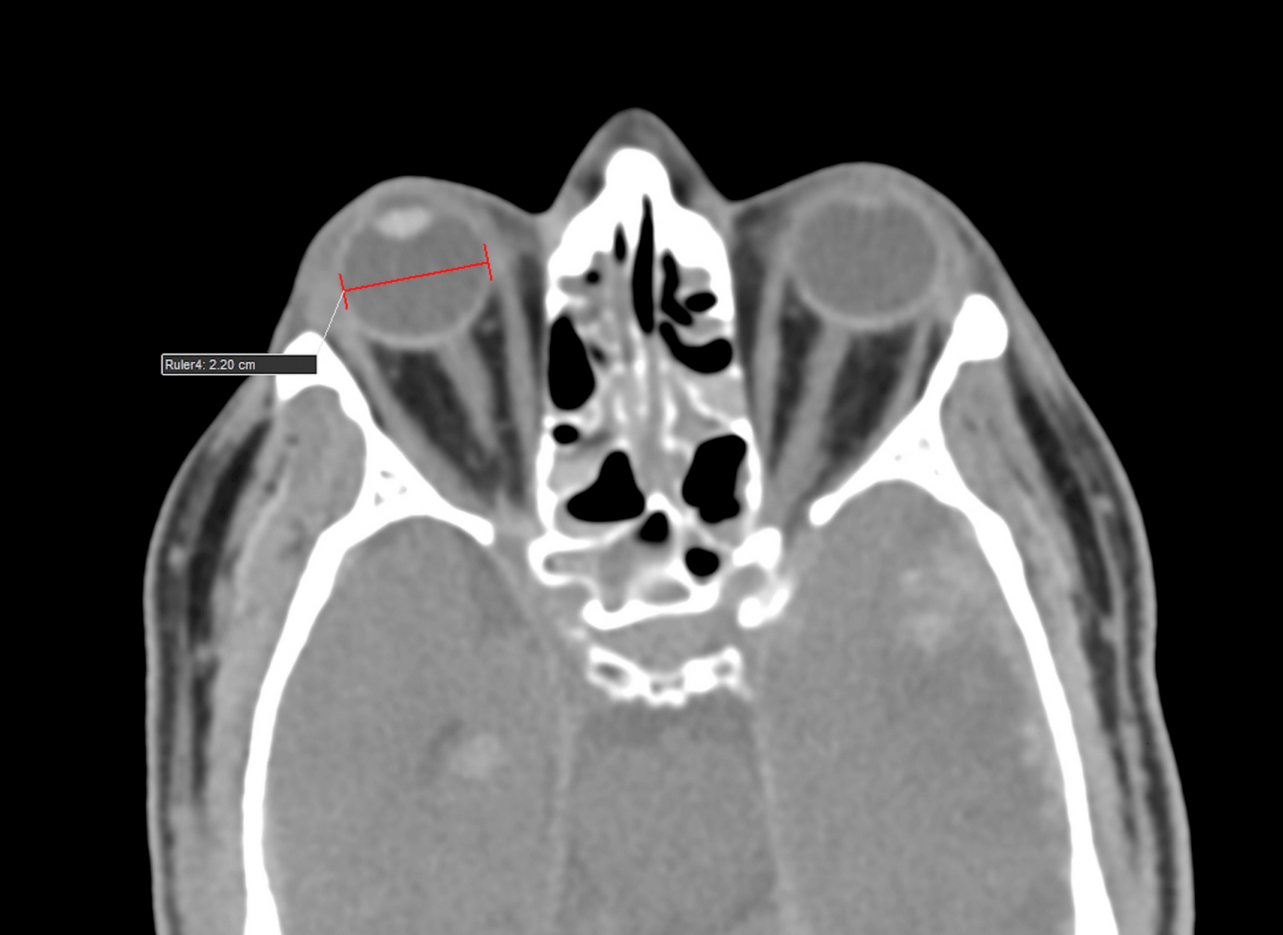
A 55-year-old male who was involved in a road traffic accident and sustained a head injury presented with a Glasgow Coma Scale (GCS) of 7. The initial computed tomography (CT) scan of the brain (axial section, 1 mm slice thickness, standard mediastinum algorithm with a window level of 10 Hounsfield units {HU} and a width of 300 HU) shows an eyeball transverse diameter (ETD) of 22 mm (from sclera to sclera), indicating severe traumatic brain injury (TBI).

A 55-year-old male who was involved in a road traffic accident and sustained a head injury presented with a GCS of 7. An initial CT scan of the brain using a 1 mm slice thickness and the standard mediastinum algorithm (with a window level of 10 Hounsfield units {HU} and a width of 300 HU) showed significant findings, with an RCTS of 5. The ONSD measured at 3 mm behind the globe was 6.5 mm. The ETD measured from sclera to sclera was 22 mm (Figures 5-7). These findings are indicative of a severe TBI.

## Discussion

This study is the first to use the ONSD-to-ETD ratio as a tool correlating with the GCS and the RCTS. We found a robust and independent correlation between the ONSD/ETD ratio and both GCS and RCTS in consecutive brain CT scans in individuals who had suffered TBI. A sequential increase in the ONSD/ETD ratio, low GCS scores, and worsening RCTS were critical predictors of TBI outcomes. In total, 308 patients (200 retrospective and 108 prospective) met the inclusion criteria. The CT-ONSD, ETD, and ONSD/ETD ratio cut-off values of >5.17 mm, <22.2 mm, and >0.21, respectively, were significantly linked with elevated ICP or ICH.

According to this study, the cut-off values for ONSD are set at <5.17 mm and >5.17 mm to indicate normal and elevated ICP or ICH, respectively. These values align with the findings of Vaiman et al., who reported that in healthy individuals, the ONSD ranges from 3.65 mm to 5.17 mm [11]. Similarly, for ETD, cut-off values are >22.2 mm and <22.2 mm, predicting normal and elevated ICP or ICH, respectively. The study by Bekerman et al. found that in healthy people, the ETD averaged 24.156 ± 1.9 mm in the right orbit (maximum of 26.8 mm and minimum of 21.5 mm) and 24.324 ± 1.9 mm in the left orbit (maximum of 27.1 mm and minimum of 20.9 mm) [12].

The ONSD/ETD ratio's cut-off values (<0.21 and >0.21) predict normal and elevated ICP or ICH, respectively, similar to Vaiman et al.'s study, which found an ONSD/ETD ratio of 0.19 ± 0.01-0.02 [11]. Similarly, Bragée et al. reported a mean ONSD/ETD of 0.24 ± 0.03 [13]. The RCTS cut-off values (2-3 for mild and ≥4 for severe) were consistent with Das et al.'s findings [14], while GCS cut-off values (>12 for mild and <12 for severe) matched those by Jain and Iverson, who classified GCS as 13-15 (mild), 9-12 (moderate), and 3-8 (severe) [15].

Chi-square and p-values for the variables between GCS (<12 and >12) and ONSD (<5.17 mm and >5.17 mm), ETD (>22.2 mm and <22.2 mm), and the ONSD/ETD ratio (<0.21 and >0.21) were 39.9 and 0.000, 2.132 and 0.153, and 18.52 and 0.000, respectively.

Correlation analysis revealed that the mean ONSD and RCTS had a strong positive correlation (0.82, p = 0.01) (two-tailed), whereas mean ETD and RCTS had a moderate negative correlation (-0.50, p = not significant). The mean ONSD/ETD ratio and GCS had a strong negative correlation (-0.783, p = 0.01) (two-tailed). Spearman's rho for GCS and RCTS are -0.931 (p = 0.000), with an R² of 0.854.

The study presents the following values in the order of sensitivity, specificity, positive predictive value, and negative predictive value: GCS: 100%, 86.2%, 44.9%, and 100%; RCTS: 100%, 74.3%, 30.3%, and 100%; ONSD: 100%, 83.7%, 40.7%, and 100%; ETD: 87.0%, 79.4%, 32.1%, and 98.2%; and the ONSD/ETD ratio: 100%, 95.6%, 72.0%, and 100%. The areas under the curve for GCS, RCTS, ONSD, ETD, and the ONSD/ETD ratio were 0.000, 0.965, 0.932, 0.490, and 0.920, respectively, demonstrating significant associations between the variables.

The eyeball's anterior to posterior diameter varies, especially in myopia, hypermetropia, and emmetropia cases. The ONSD correlates with the eyeball size, which can be advantageous. The optic nerve/eyeball diameter index is less variable for elevated ICP monitoring. The index calculation is as follows: we measured the ONSD from the distal part of the optic nerve's intra-orbital segment and divided it by the eyeball's transverse diameter (ONSD/ETD). We selected the ETD because most authors measured the ONSD in the transverse plane for ICP monitoring.

ICH leads to the pathophysiology of optic nerve sheath enlargement. The ONSD measurement is based on images taken from a constantly moving object using modalities such as ultrasonography (USG), CT, and magnetic resonance imaging (MRI). We cannot ignore other eye movements, such as constant physiological tremors, tracking movements, smooth pursuits, slow drifts, flicking movements, and saccades. The optic nerve's total axon count decreases, the mean axon diameter increases, and the dura mater thickness increases with age [16]. Although all these processes coincide, the ONSD remains unchanged over a lifetime. Some data indicate that optic nerve fiber loss increases with age [17].

Zinn and Wrisberg republished "Descriptio Anatomica Oculi Humani" in 1780, noting that the dura mater provides a sheath that encases the optic nerve [18]. Cranial meninges (subarachnoid space) cover the optic nerve sheath and bathe it in cerebrospinal fluid. The arachnoid trabeculation's fibrillary arrangement and density are responsible for optic nerve sheath enlargement. The ONSD's expansion indicates elevated ICP transmission to the intra-orbital perineural subarachnoid space [19].

The right and left ONSD at 3 mm and 8 mm from the globe and 3 mm from the optic canal were 4.94 ± 1.51/5.17 ± 1.34 mm, 4.35 ± 0.76/4.45 ± 0.62 mm, and 3.55 ± 0.82/3.65 ± 0.7 mm, respectively [20]. Helmke and Hansen demonstrated the importance of selecting an identical distance for the optic nerve sheath measurement in 1996 [21]. The ONSD, 3 mm from the eyeball, showed broader changes than other optic nerve segments. In normal subjects, the subarachnoid space is most expansive behind the eyeball and then tapers toward the orbital apex. At rest, the eyeball experiences voluntary and involuntary movements while the optic nerve's head remains in motion.

Researchers have used neuroimaging techniques to look at radiological markers to assess the severity and prognosis of TBI. In particular, CT is the gold standard imaging modality for detecting fractures and hematomas in patients with TBI. CT is divided into two classification systems to assess TBI severity: (a) the Marshall CT and (b) the RCTS. The Marshall classification does not consider subdural, epidural, or subarachnoid hemorrhages, while the RCTS is an independent predictor of TBI outcomes [22,23]. Multiple studies have proven that a high RCTS is associated with a raised mortality rate.

The ONSD independently predicts TBI morbidity and mortality [24]. Imaging methods that accurately estimate the ONSD include CT, MRI, and USG. Recently, ONSD measurement has used USG or CT as a noninvasive method of monitoring ICP and, consequently, the severity of brain injury [25]. In the literature, the association between ETD and clinical parameters remains unclear. Calculating the ONSD/ETD ratio helps to eliminate the variability and bias caused by interocular anatomical size variations. Papilledema, or optic disc swelling, is an ophthalmoscopic diagnosis that indicates a raised ICP without ocular pathologies. USG and ophthalmoscopy evaluate the optic nerve head and disc with high sensitivity but low specificity, subjectivity, and interobserver variation.

We recommend the USG measurement of the ONSD/ETD ratio due to its safety, lack of ionizing radiation, portability, and ability to provide immediate results in the ICU and ER at the point of care. However, USG has disadvantages, such as dependency on skilled personnel and potential delays in diagnostics due to human resource logistics.

Global guidelines for head injuries measure the ONSD/ETD ratio using a CT scan. This method gets extra information from a rule-out CT brain study without exposing the patient to more ionizing radiation. The CT measurement of the ONSD/ETD ratio also offers objectivity, simplicity, and near-real-time reflectivity for raised ICP. However, CT has disadvantages, including high costs and cumulative radiation exposure. Nevertheless, the clinical benefits far outweigh the associated radiation risks.

The MRI measurement of the ONSD/ETD ratio has the advantage of high spatial resolution and, thus, high objectivity. However, MRI's disadvantages, such as its high cost, lack of universal availability, relatively longer scan times, susceptibility to motion artifacts, and subsequent image degradation, limit its use in acute TBI settings. Maas et al. highlighted the pitfalls of the Marshall CT classification and reintroduced the RCTS [26]. The RCTS, expressed in numerical values from 1 to 6, predicts outcomes [27].

Different authors use different techniques for measuring CT-ONSD; some measure 3 mm from the eyeball, whereas others measure 10 mm [28]. The optic nerve sheath is more extensible because of its anterior region's small number of trabeculations. Stretched optic nerve sheath distension, a sign of high ICP, causes papilledema. Without a fundoscopy, papilledema takes hours or days to appear. Papilledema is one of the standards for diagnosing idiopathic ICH, per the modified Dandy criteria [29].

In TBI, CT is the routine imaging modality. The RCTS and ONSD are independent parameters that predict outcomes in TBI, and they are much less variable than the ONSD for ICP monitoring. In this regard, we aimed to evaluate the ONSD/ETD ratio measurement on the sequential thin-slice CT scan in correlation with the GCS and RCTS. We used the RCTS to assess the severity and prognosis of TBI based on the imaging findings. The RCTS ranges from 1 to 6, with a higher score indicating a more severe head injury. A timely diagnosis of raised ICP secondary to a TBI suggests a poor prognosis and is crucial for management.

Limitations of the research

Despite the limitations of retrospective data collection and the disadvantages of using CT for measuring ONSD, this study provides valuable insights into establishing the ONSD/ETD ratio in sequential CT scans of TBI patients. These insights could improve patient care by enhancing our understanding of the correlation between the ONSD/ETD ratio, GCS, and RCTS parameters.

No other study has yet analyzed the potential correlation between the ONSD/ETD ratio and the GCS and RCTS, primarily due to the absence of comparative literature. However, our analysis upholds the availability and performance of invasive ICP monitoring. Invasive ICP monitoring remains a challenge in developing countries, where ICP monitors may be unavailable or prohibitively expensive. Since a single investigator performed all measurements, assessing the method's interobserver and intraobserver variability was impossible. Despite these limitations, we highlight the necessity for further studies that report the ONSD/ETD ratio in patients undergoing sequential CT scans as part of the imaging protocol for TBI.

Another limitation of this research is the potential for differences in optic nerve dimensions and eyeball size between different ethnic backgrounds. The study did not find significant discrepancies in ONSD and ETD among hospitalized patients of various ethnic groups. Research from China suggests that the anterior segment of Asian eyes may be smaller than that of Caucasian eyes [30]. The ONSD ranges from 3.55 ± 0.82 mm to 5.17 ± 1.34 mm in various locations within the intra-orbital space, and there is no significant difference between age groups and genders in healthy individuals.

Key messages

The CT-ONSD/ETD ratio is associated with the GCS and RCTS in TBI and can indicate patient outcomes. However, it is essential to note that a high ONSD/ETD ratio does not necessarily exclude the possibility of a favorable outcome. Given the widespread availability of CT scans in ERs, measuring the ONSD/ETD ratio may assist clinicians in making informed decisions regarding transferring patients to specialized referral centers. Most CT consoles have digital tools for quickly calculating the ONSD/ETD ratio. Furthermore, integrating the ONSD/ETD ratio with other clinical, radiological, and laboratory parameters could enhance the management of TBI patients.

## Conclusions

Using CT to find the ONSD-to-ETD ratio seems like a practical, noninvasive way to measure ICP in people who have had a TBI. Based on known severity markers such as the GCS and RCTS, our results show statistically significant connections between the ONSD/ETD ratio. A steady rise in the ONSD/ETD ratio, low GCS scores, and worsening RCTS are all signs that critical ICP is developing in trauma settings. While a high ONSD/ETD ratio is associated with poor outcomes, it is crucial to note that it does not necessarily preclude the possibility of favorable outcomes. Given the increasing availability of CT scans in ERs, we suggest including the readily calculated ONSD/ETD ratio in TBI patients' admission reports to support severity assessment, ICP change monitoring, and well-informed decision-making regarding patient transfers to specialized care centers. Furthermore, it integrates the ONSD/ETD ratio with other clinical, radiological, and laboratory parameters for comprehensive patient evaluation and management. Future research should focus on multicenter validation analyses and investigations of applications in various clinical settings to demonstrate the role and benefit of the CT-ONSD/ETD ratio in improving triage, monitoring, and outcome prediction for TBI patients.
